# The times, movements and operational efficiency of mechanized coffee harvesting in sloped areas

**DOI:** 10.1371/journal.pone.0217286

**Published:** 2019-05-28

**Authors:** Tiago de Oliveira Tavares, Bruno Rocca de Oliveira, Vantuir de Albuquerque Silva, Rouverson Pereira da Silva, Adão Felipe dos Santos, Estela Silva Okida

**Affiliations:** Department of Agricultural Engineering, Laboratory of Agricultural Machinery and Mechanization, São Paulo State University, Jaboticabal, São Paulo, Brazil; Potsdam Institute for Climate Impact Research, GERMANY

## Abstract

Coffee farms have been adopting the microterraces system, a technique that reduces the effect of the slope by moving the soil between the crop lines. In this way, all the mechanized operations can be carried out normally, except for harvesting, due to the work limitation of the harvesters, who work in areas with a maximum slope of 20%. One option is to use unilateral harvesters, which crop one side at a time; however, there has been no research on these microterrace machines to evaluate their performance and to compare it with those of the other harvesting methods in those regions. This study aimed to compare the mechanized harvest performance in the microterraces with the manual and semimechanized harvesting methods. The study was carried out in an agricultural area of the municipality of Ouro Fino / MG, Brazil, in a crop production site where the microterraces were built six years before the experiment. The treatments were assigned to a split-block design with seven repetitions and consisted of mechanized harvest—unilateral harvester with bag storage; manual harvest—regionally experienced workers; and semimechanized harvest—with portable breakers. Through an analysis of the times and movements, the operational efficiency and operational and effective field capabilities were measured. The adoption of microterraces allows the efficient mechanization of areas previously impossible to mechanize. The unilateral harvester is a potential tool for the partial replacement of manual labor in the harvest, performing a service equivalent to that of 23.68 manual workers and 10.55 manual workers in the semimechanized system.

## Introduction

Coffee is one of the main crops produced in Brazil and is mainly concentrated in the southeast region. The domestic production of coffee accounts for 30% of the world’s production and is the sum of the other six major world producers; thus, coffee is important for the Brazilian economy. In addition, Brazil is the second largest consumer market behind the United States [1].

A commonly adopted production system is the "zero crop" system, whose purpose is to reduce costs by reducing the size of the plants by pruning them during years of low coffee productivity, with the aim of increasing production for harvest in a later year [2]. The plants are harvested during the year of "zero crop", and their canopies are quickly recomposed [3].

Agricultural terraces are quite common in mountainous regions and contribute to long-term production [4]. The practice has attracted the interest of researchers because it aims at sustainability and an extremely well-structured management [5].

The construction of terraces has been used in several crops, especially those that demand good soil quality without water loss, such as grape plantations in Europe [6]. The use of terraces is implemented in several regions of the world with the aim of managing and conserving the soil and increasing the flow and the rate of infiltration, thus reducing the concentration of water in the soil, and is related to the amount of vegetal cover in the soil [4].

Studies have shown that coffee cultivation in mountainous regions shows better productivity [7] when associated with good levels of rainfall in the rainy season, for example, in the summer [8]. To increase the speed and quality of the harvest in these regions, mechanized harvesting and semimechanized harvesting have been increasingly used [9].

A relatively new technique in coffee cultivation, terracing is a potential solution that limits mechanization in these mountainous areas [10]. These terraces are up to 1.6 meters wide and are sufficient for the entry of coffee and compact tractors [7].

The extent to which the marked slope can reduce the operational capacity of the tractor and the time and energy demanded in the harvesting operation still need to be addressed [11]. According to [12], without terracing, slopes higher than 20% can require 21.6% more time than that required on lower slopes, which is mainly due to necessary maneuvers. Because microterraces have been gaining importance, the shortage of manpower in mountainous regions makes mechanization an important strategy for producers of coffee.

Because terracing is an efficient and sustainable tactic, farmers in the southern region of Minas Gerais-Brazil have recently adopted the terrain between the lines of the mountain for coffee culture. This structure allows mechanization to be assisted on high slopes to reduce costs and make the harvest more efficient. However, no study has evaluated the performance of the machines that are adapted to operate in this situation. Thus, assuming that the use of microterraces for mechanized harvesting of coffee can increase the harvesting efficiency by reducing the operation time, the goal of this work was to compare the efficiency of mechanized harvest with that of manual and semimechanized harvest (use of portable harvesters).

## Materials and methods

No specific permissions were needed to access the experimental sites because all activities were performed under the supervision of the advisor of the thesis, and did not involve threatened or protected species.

### Experimental site

This work was carried out at Fazenda Vale Verde, in the municipality of Ouro Fino—MG, located near 22°14'S and 46°20'W, with an average elevation and slope of 1036 m and 47%, respectively. The soil of the site has a clay-like texture and is classified as ALUMINUM according to the classification of [13]. The climate is Cwb according to the classification of Köppen [14], with an average precipitation of 1518 mm annually. Due to the high slope of the area, cultivation is carried out on terraces, as illustrated in S1 Fig.

The experimental area corresponds to 10.5 ha with level lines, and the Catuaí Amarelo 99 variety, approximately 40 years of age, is cultivated at a level of 3.25 m between the rows and 1.1 m between the plants (2.797 plants ha^-1^) without irrigation. The crop under study was subjected to the zero crop system and was harvested every two years due to pruning. The zero crop system consists of a cyclical pruning of the coffee tree, trimming the tree like a skeleton (lateral pruning), followed by cleavage (upper pruning) in the year of production. This practice is characterized by the pruning of plagiotropic branches of the plant, with a distance of 20 to 30 cm from the orthotropic branch at the top and ending at the bottom with a cut distance of 30 to 50 cm. This system aims, through programmed pruning, to obtain high yields.

### Treatments

The treatments consisted of coffee harvesting using three methods. Two methods are common in the mountainous regions: manual harvesting (T1) and semimechanized harvesting (T2) with portable crushers; finally, a new method was proposed using mechanized harvesting (T3) with a harvester designed for microterraces. The treatments were assigned to a split-block design with seven repetitions.

Before harvesting, the degree of ripening of the fruits was evaluated at eight random points, and the fruits were separated into greens, cherries and dried raisins. At the time of harvest, there were 21.59, 30.23 and 48.17% green fruits, cherries and raisins, respectively. The average productivity of coffee was 68.57 bags ha^-1^ (4114.20 kg ha^-1^), and 27.09% of the crop was on the ground due to the occurrence of atypical rains in the months of May and June, which caused the fruits to detach prematurely from the branches.

The manual and semimechanized harvesting were performed with two experienced workers harvesting a total area of 420.0 m^2^ using plots 10 m long covered with raffia canvas placed on the ground under the skirt of the coffee, denoted the “harvesting canvas” (S2 Fig), which was finally converted proportionally to ha. The times were measured in each of the seven plots for each treatment for the all operations performed (times for changing harvesting canvas, harvesting, cleaning and transportation). With this method, it was possible to examine the amount of coffee harvested and the total load of coffee in L ha^-1^. Semimechanized harvesting was performed using a STIHL KA 85 R SP multifunctional portable harvester.

For the mechanized operation, a J-FLEX unilateral harvester (model FMA 079/15) was drawn by a Valtra BF 75 4x2 FWD tractor operating at a speed of 0.7 km h^-1^ as shown in S3 Fig. The harvester had 33 sets of 18 stems, with a total height of action (distance between the first and last set) of 1.87 m; the stems were 50 cm long and 6 cm apart (distance between the sets); and the hydraulic adjustment for the insertion height rose up to 1.3 m from the ground. The belt of the pickup system was of the conventional type with a width of 20 cm and a length of 1.65 m at the top. The brake had a resistance of 4.5 kg, which was regulated for cultivating plants with a high degree of maturity. In this method of harvesting, an area of 9,544.9 m^2^ was used for evaluation. The times were measured in each of the seven plots for the all operations performed (times for harvesting, maneuvering, unloading and bag change) and converted proportionally to ha.

The times and movements of the three evaluated treatments were timed and characterized according to S1 Table. Measurements were taken during harvest considering the areas described previously.

### Experimental design and statistical analysis

The quantities of coffee in L ha^-1^ were divided into the following groups: present on the ground before harvest, harvested, remaining (fruits on the tree after the harvest) and fallen from the machine. The total load was given by the sum of the four previous groups.

To compare the performances of the harvesting methods, the time and movement methodology were used according to the following criteria:

- Time spent on each harvest method, evaluating the quantities of harvested plants, area in m^2^, estimation of plants ha^-1^ and manual, semimechanized and mechanized harvesting operations.
- Amount of coffee in L ha^-1^ from manual, semimechanized and mechanized harvests (quantity on the ground before, quantity of harvested coffee, remaining quantity, quantity dropped in the machine and total load).
- Decrease in weight in g plant^-1^ for each type of crop.

After the data acquisition, the times obtained were extrapolated to the area of one hectare. The operational efficiency was calculated according to [15], while the time efficiency and the operational and effective field capacities were determined according to [16].

The effective field capacity was adapted from [16] and calculated by means of Eq 1:

$$EfC=\frac{Hs\times S}{10}$$ (1)

where

*EfC* is the effective field capability (ha h^-1^);

*Hs* is the harvest speed (km h^-1^);

*S* is the spacing between the coffee lines (m);

and 10 is the factor of suitability of the units.

The operational field capacity was adapted from [17] according to Eq 2:

$$OfC=\frac{Hs\times S\times Ef}{10}$$ (2)

where

*OfC* is the operational field capacity (ha h^-1^);

*Hs* is the harvest speed (km h^-1^);

*S* is the spacing between the coffee lines (m);

*Ef* is the efficiency of the harvester;

and 10 is the factor of suitability of the units.

The efficiency of the harvester represents the percentage of the time in which it is actually in operation, discounting the maneuvers and the discharge versus the total time (Eq 3):

$$\mathrm{Ef}=\{\frac{Ht}{\mathrm{Ht}+Ot+Dt}\}\;x\;100$$ (3)

where

*Ef* is the efficiency of the harvester (%);

*Ht* is the harvesting time (s);

*Ot* is the operating time (s);

and *Dt* is the download time (s).

The results were analyzed by the AgroEstat–System for Statistical Analysis of Agronomic Tests and were compared by the Tukey test at 5% probability.

## Results and discussion

S2 Table shows that the operational efficiency (time efficiency) of the treatments varied from 61.54 to 79.93% and that the mechanized and manual harvests presented the best averages in relation to the semimechanized harvest, which presented a decrease in operational efficiency on the order of 17.84% and 20.01% when compared to those of the manual and mechanized harvests, respectively. The lowest average operational efficiency observed in the semimechanized harvesting occurred due to the actual harvesting time, which was reduced due to the faster fruit rotting, and the time spent changing the harvesting canvas, cleaning and transporting the coffee had a lower yield per time, which was equivalent to that of the manual harvest.

According to [18], the ideal operational efficiency is 71.94% for a mechanized wheel with large implements; therefore, in this work, the result was considered acceptable, and the variations in operational efficiency were justifiable according to the slope and size of the plots. Additionally, the present study indicates that the mechanized harvest obtained an operational efficiency average of 76.93% with the use of microterraces and smaller machines; in the case of the J-FLEX, which stabilizes the speed and slope of the terrain, [19] stated that the values of operational efficiency that are considered acceptable are between 70 and 90%.

According to S4 Fig, manual harvesting occupied 75% of the harvesting time (productive time), and the rest of the time was spent on complementary activities: 7% for changing the harvesting canvas, 16% for cleaning and 2% for transporting the coffee for the carriers. In the semimechanized method, the productive time was 61%: 13% for changing the harvesting canvas, 19% for cleaning and 7% for transporting; the factor is expressed in the results of EfC and OfC (S3 Table).

In mechanized harvesting, 78% of the total time was spent on harvesting, 10% on maneuvering, 3% on positioning the bag in the back of the machine during auxiliary stops (straightening) and 9% on transporting the bag to the carrier. The maneuvering at mechanized coffee harvesting in mountains is hard and can be a problem for farmers because when the machine came on the crop line end, the operator needs more time to make maneuvering due the high slope, which decreasing operational efficiency.

The proportions of harvesting times were higher in semimechanized and manual harvesting, which makes the operational efficiency of the two evaluated treatments lower than those in mechanized harvesting; however, the percentage of each process was different, indicating significant differences (S2 Table).

The effective field capacity is the ratio of the number of hectares harvested in a given period, and thus, S3 Table shows that the mechanized harvest operation presented superior results in relation to those of the semimechanized and manual operations; the effective field capacities of the mechanized, semimechanized and manual operations were 0.11 ha h^-1^, 0.01 ha h^-1^ and 0.01 ha h^-1^_,_ respectively.

The mechanized harvesting operation obtained better results with shorter times in relation to the amount of the harvested area; in a time of 1.36 hours, the mechanized harvesting operation was carried out in 0.81 ha compared to the 0.04 ha harvested using the semimechanized operation and the times of 0.69 hours and 1.70 hours for the semimechanized and manual operations, respectively.

Manual harvesting requires a longer time than mechanized harvest of coffee, and these values are lower even with the necessary transfer operations in the mechanized harvest; [20] the average effective times of harvest were 50 min 20 sec for manual harvesting and 26 min 40 sec for mechanized harvesting, showing that mechanized harvesting is faster.

According to [10], the best operational capacity of 0.018 ha h^-1^ was reached with the use of another machine for mountain coffee growing; in the case of a prototype assembled by [10], in addition to this value, [21] also obtained 0.015 ha h^-1^, a lower value than that of the mechanized harvest obtained in the J-FLEX experiment, which was 0.0831 ha h^-1^.

The operational field capability shows that, among all the harvesting operations described for the effective field capacity, the mechanized harvest has the largest OfC and that its harvest times including all maneuvers were better than those of the two treatments mentioned (S3 Table).

The higher efficiency of the mechanized harvesting demonstrates that a machine is capable of replacing a specific workforce (S4 Table), with manual harvesting requiring 35.65 men on day 1 to harvest 1 hectare. In the semimechanized method, 15.88 men day^-1^, compared to the mechanized harvest that was used for 1.51 days, was necessary to harvest the same hectare with the unilateral harvester. Although high slope coffee harvesting—whether manual or mechanized—is carried out on each side of the plant at a time, the mechanized harvesting is more efficient because it is able to engage the harvesting processes (fruit detachment, cleaning, separation, and transportation) with greater speed.

In addition to displaying lower values of the services in ha^-1^, the efficiency of the harvester corresponds to 23.68 people harvesting manually and 10.55 people with a portable harvester (S2 Table). [22] states that mechanization in the harvesting process reduces the cost of harvesters per sack since the rapid perishability of coffee influences the costs, with the mechanized harvest having lower losses due to fruit maturation.

S5 Table shows that the operations of manual harvesting and the use of the breakers did not differ statistically, with the volume of coffee harvested per hectare being greater than that harvested by the J-FLEX. This fact can be explained by the management of each type of harvest: in a manual harvest, there are fewer failures, and it is possible for a worker to ensure that the fruits present in the plant are removed and that no remaining coffee is observed. In contrast, the disadvantage is the duration of each treatment, with the mechanized harvest being the fastest.

According to [23], the harvester is capable of harvesting 14,268.56 L ha^-1^, equivalent to 5.9 L per plant on smaller slopes, a value that is higher than that evaluated with J-FLEX, which is capable of harvesting 11,059 26, L ha^-1^, equivalent to 4.57 L plant^-1^.

The difference is due to the terrain, where on the high slopes, the area reached in each plant is smaller; nevertheless, the results are satisfactory in relation to the terrain, where the difference is 1.33 L plant^-1^. However, the possibility of mechanization on slopes above 40% increases the yield of the harvest, which is advantageous in spite of the differences.

The mean values of the remaining coffee were not presented for the semimechanized and manual treatments because these were manual harvests; in the mechanized harvesting operation, it was observed whether there was a need for a pass-through on each plant. For this reason, the two treatments did not differ statistically since the mechanized harvest was differentiated in order to expose the need to pass through (S6 Table).

In [24], 8 different progenies were evaluated, with a mean of 8165.74 L ha^-1^ of fruits remaining on the plant. In contrast, J-FLEX used on a highly sloped terrain with microterraces obtained an average value of 7760.23 L ha^-1^ of coffee remaining on the plant, demonstrating the advantage of the use of microterraces for mechanized harvesting.

One advantage of passing the remaining coffee is a higher quality harvest; in the interval from one operation to another, the green fruits ripen to cherry fruits, increasing the selectivity [25].

The amount of coffee dropped on the ground, given in L ha^-1^, was noted only for the mechanized harvesting operation; therefore, the results of the operations using the harvester and of the manual operation did not differ statistically because they were nonexistent (S7 Table).

The mechanized operation causes the coffee to fall to the ground due to the crop structure; when passing by the plant with the J-FLEX, the fruit falls directly in the bag; in this way, some fruits end up falling on the ground, followed by the need to sweep. The semimechanized and manual operations have a different structure, in which the harvested coffee falls directly onto a harvesting canvas 10 meters long and wide enough to cover the breadth of the street; thus, the largest harvest area has the least chance of the coffee landing on the ground.

[25] obtained a mean of 1225.83 L ha^-1^ of coffee fallen in the mechanized harvest, and this value is more promising due to the difference in the reach of the plant in the mechanized harvest; in the present work, the amount of coffee dropped was 4545.06 L ha^-1^.

Plant defoliation may lead to a decrease in the fruiting of the coffee tree by lowering the reserves necessary for the formation of the fruits, which consequently decreases its production [26]; therefore, it is important to evaluate the amount of defoliation present in each of the operations.

From S8 Table, a greater amount of defoliation can be observed in the manual harvest, which is significantly different from that in the mechanized harvest; however, the semimechanized harvest did not differ statistically from the other two treatments. [27] obtained higher leaf stripping results than when the J-FLEX was used in the mechanized harvest, which achieved 540 g plant^-1^ compared to 259.08 g plant^-1^ that was achieved in the present experiment.

The manual harvest had the highest defoliation value of 525.75 g plant^-1^, followed by the semimechanized harvest with a value of 481.33 g plant^-1^; finally, the mechanized harvest presented the lowest defoliation index, at 265.34 g plant^-1^. In fact, previous studies have reported that mechanized harvesting damages the plant less than manual harvesting or harvesting with the use of portable devices [28].

## Conclusions

Despite the need for transferring and sweeping during the harvest and postharvest operations, respectively, mechanized harvesting is preferable, even in sloping areas, because it presents a significantly higher operational capacity than the semimechanized and manual treatments. The reduced time and increased efficiency will improve other operations.

The defoliation is lower in mechanized harvesting, thus reducing future losses in plant production and presenting a more efficient harvesting timing.

## Supporting information

S1 Fig Cultivation of coffee on terraces; 47% slope; Ouro Fino–Minas Gerais, Brazil. (DOCX)

S2 Fig Semimechanized harvesting with a multifunctional portable harvester. (DOCX)

S3 Fig Coffee harvester used for high slopes; J-Flex. (DOCX)

S4 Fig Description of the times spent in each type of harvest. Manual harvesting (a), semimechanized harvesting (b) and mechanized harvesting (c).
(DOCX)

S1 Table Time and movements in the manual, semi-mechanized and mechanized harvesting of microterraced coffee. (DOCX)

S2 Table Mean values of operational efficiency. (DOCX)

S3 Table Average values of effective field capacity and operational field capacity in ha h^-1^. (DOCX)

S4 Table Average of services ha^-1^ of all treatments. (DOCX)

S5 Table Mean values of the amount of coffee harvested, in L ha^-1^. (DOCX)

S6 Table Mean values of the amount of coffee remaining on the plant, in L ha^-1^. (DOCX)

S7 Table Average amount of coffee dropped on the ground, in L ha^-1^. (DOCX)

S8 Table Average weight of defoliation in the three treatments, in g plant^-1^. (DOCX)
