# Supplementary figures and images for: The times, movements and operational efficiency of mechanized coffee harvesting in sloped areas
Source: PLoS One. 2019 May 28;14(5):e0217286. doi: 10.1371/journal.pone.0217286 (PMC6538159; doi:10.1371/journal.pone.0217286)

| 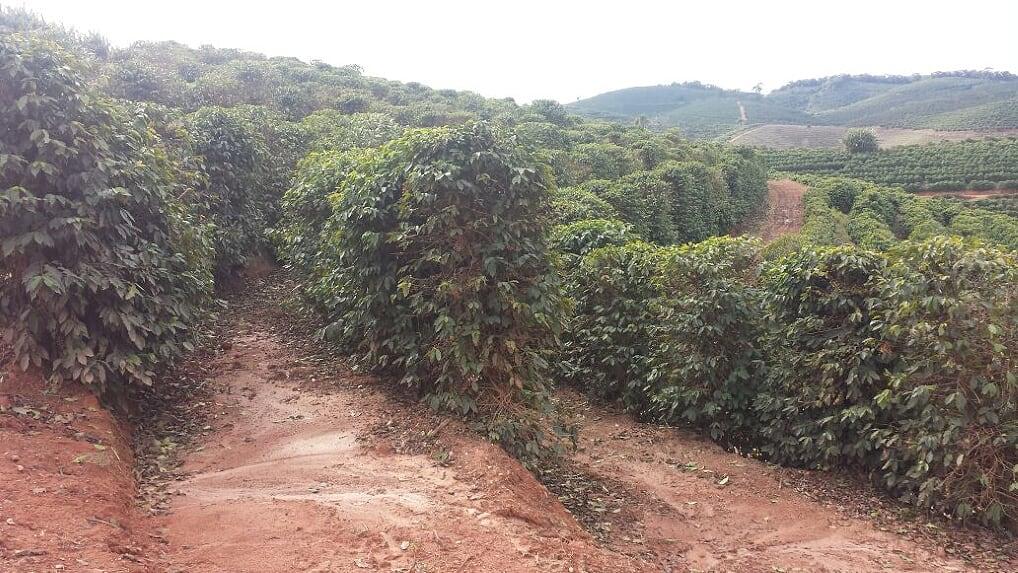 |
| --- |
| **S1 Fig. Cultivation of coffee on terraces. 47% slope. Ouro Fino – Minas Gerais, Brazil.** |

Supplement: S1 Fig — (DOCX) [file pone.0217286.s001.docx]

| 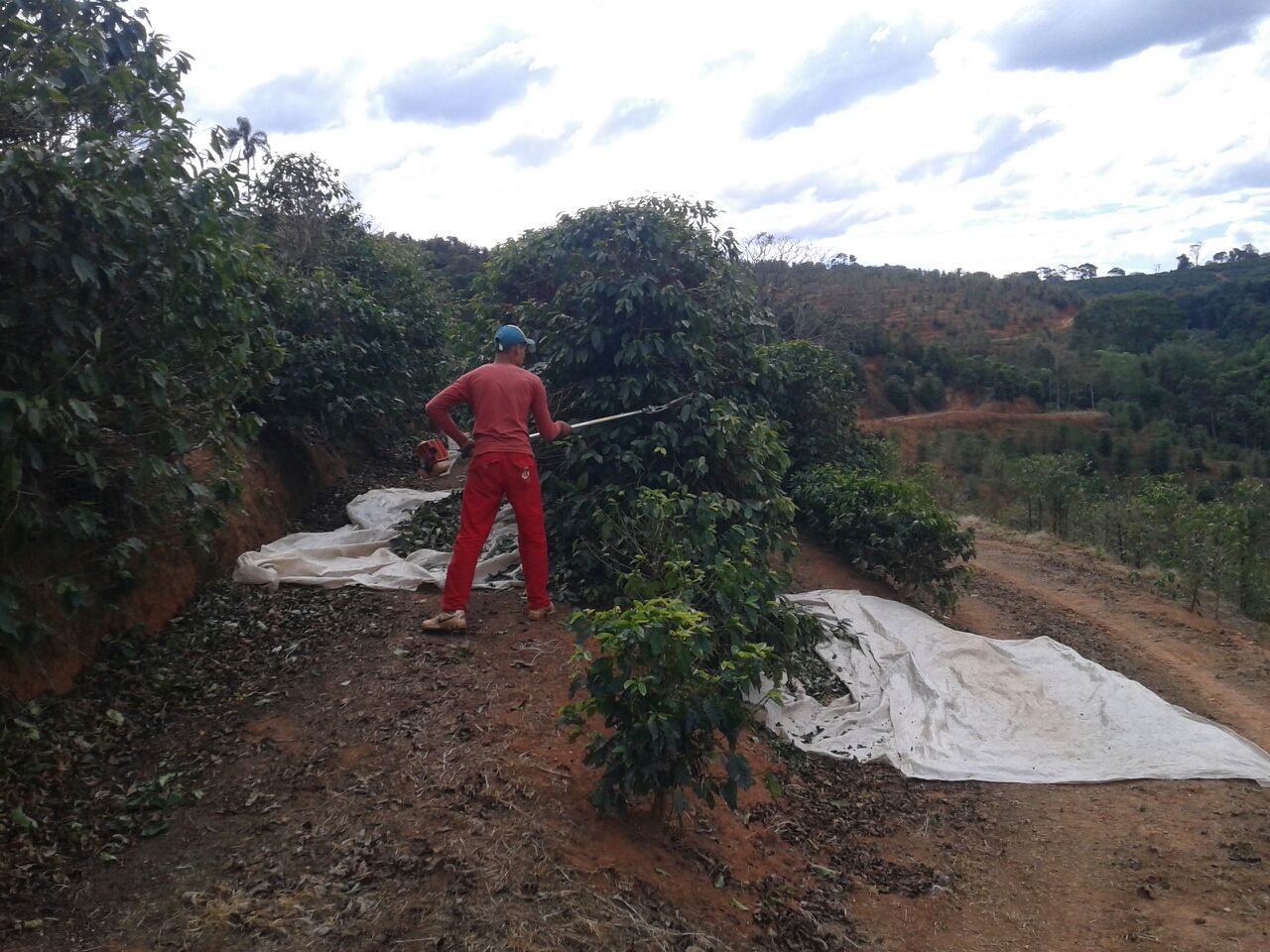 |
| --- |
| **S2 Fig. Semimechanized harvesting. Multifunctional portable harvester.** |

Supplement: S2 Fig — (DOCX) [file pone.0217286.s002.docx]

| 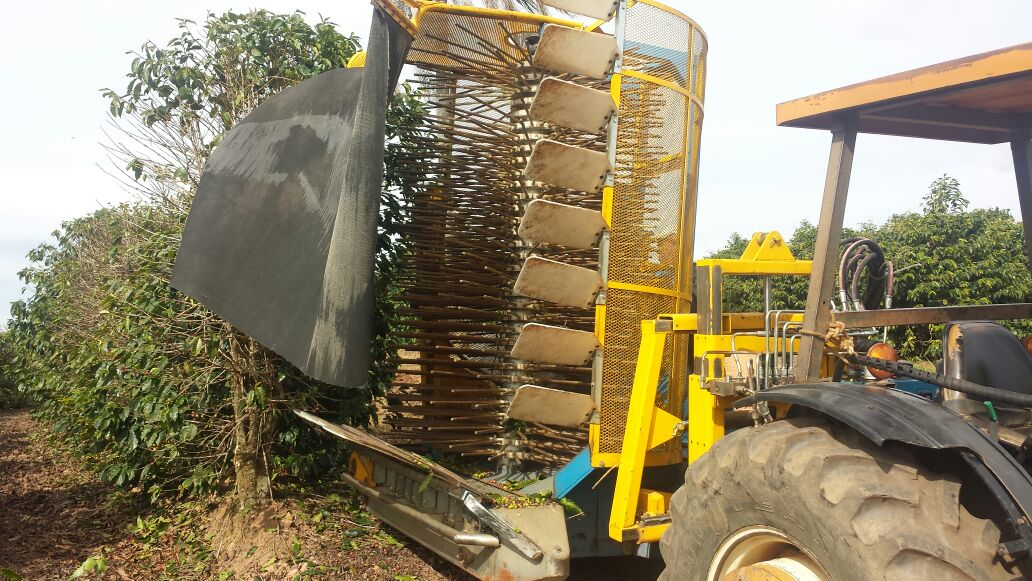 |
| --- |
| **S3 Fig. Coffee harvester used for high slopes. J-Flex.** |

Supplement: S3 Fig — (DOCX) [file pone.0217286.s003.docx]
