# Supplementary material for: The times, movements and operational efficiency of mechanized coffee harvesting in sloped areas
Source: PLoS One. 2019 May 28;14(5):e0217286. doi: 10.1371/journal.pone.0217286 (PMC6538159; doi:10.1371/journal.pone.0217286)
Supplement: S4 Fig — Manual harvesting (a), semimechanized harvesting (b) and mechanized harvesting (c). (DOCX) [file pone.0217286.s004.docx]

| 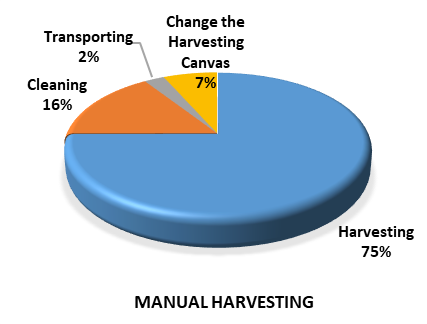  (a) | 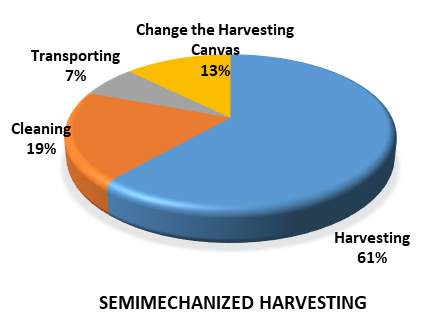  (b) |
| --- | --- |
| 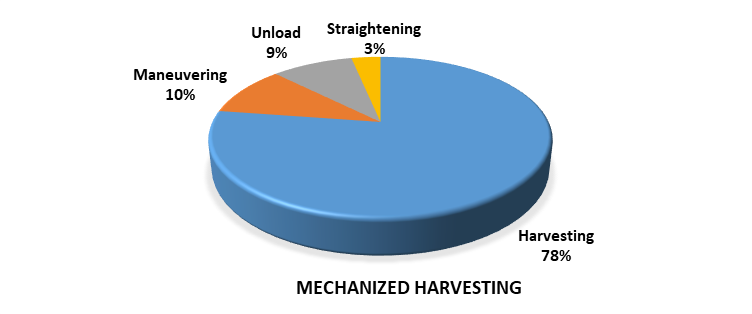  (c) | |
| **S4 Fig. Description of the times spent in each type of harvest. Manual harvesting (a), semimechanized harvesting (b) and mechanized harvesting (c).** | |
