# Supplementary material for: The times, movements and operational efficiency of mechanized coffee harvesting in sloped areas
Source: PLoS One. 2019 May 28;14(5):e0217286. doi: 10.1371/journal.pone.0217286 (PMC6538159; doi:10.1371/journal.pone.0217286)
Supplement: S1 Table — (DOCX) [file pone.0217286.s005.docx]

**S1 Table. Time and movements in the manual, semi-mechanized and mechanized harvesting of microterraced coffee.**

| TIMES | MOVEMENTS | TYPE OF HARVEST |
| --- | --- | --- |
| Harvesting | Machine performing mechanized harvesting operation itself. | Mechanized |
| Maneuvering | Machine performing maneuvers to change harvest lines. |  |
| Unloading | Machine moving to the unloading points, unloading the bags and returning to the field. |  |
| Straightening | Machine stopping to replace the full bag with an empty bag or to reposition the bag. |  |
| Harvesting | Worker harvesting the coffee present in the plant on the harvesting canvas. | Semimechanized |
| Cleaning and separate the coffee | Worker cleaning and separating the coffee fruits of the vegetable impurities. |  |
| Changing the harvesting canvas | Worker changing the harvesting canvas in the harvested area to the next plot. |  |
| Transporting | Worker transporting the harvested coffee to the bag in the carrier. |  |
| Harvesting | Worker harvesting the coffee fruits manually in the harvesting canvas. | Manual |
| Cleaning and separate the coffee | Worker cleaning and separating the coffee fruits of the vegetable impurities. |  |
| Changing the harvesting canvas | Worker changing the harvesting canvas in the harvested area to the next plot. |  |
| Transporting | Worker transporting the harvested coffee to the bag in the carrier. |  |
