# Supplementary material for: The times, movements and operational efficiency of mechanized coffee harvesting in sloped areas
Source: PLoS One. 2019 May 28;14(5):e0217286. doi: 10.1371/journal.pone.0217286 (PMC6538159; doi:10.1371/journal.pone.0217286)
Supplement: S2 Table — (DOCX) [file pone.0217286.s006.docx]

**S2 Table. Mean values of operational efficiency.**

| **Treatments** | **Operational efficiency (%)** |
| --- | --- |
| Mechanized (J-FLEX) | 76.93 a |
| Semimechanized (Breaker) | 61.54 b |
| Manual (2 workers) | 74.84 a |
| **Coefficient of Variation (%)** | **5.07** |

*Mean values followed by the same letter do not differ statistically at 5% significance according to the Tukey test.
