# Supplementary material for: The times, movements and operational efficiency of mechanized coffee harvesting in sloped areas
Source: PLoS One. 2019 May 28;14(5):e0217286. doi: 10.1371/journal.pone.0217286 (PMC6538159; doi:10.1371/journal.pone.0217286)
Supplement: S3 Table — (DOCX) [file pone.0217286.s007.docx]

**S3 Table. Average values of effective field capacity and operational field capacity in ha h^-1^.**

| **Treatments** | **EfC (ha h^-1^)** | **OfC (ha h^-1^)** |
| --- | --- | --- |
| Mechanized (J-FLEX) | 0.1076 a | 0.0831 a |
| Semimechanized (Breaker) | 0.0136 b | 0.0084 b |
| Manual (1 worker) | 0.0049 b | 0.0036 b |
| **Coefficient of Variation (%)** | **20.99** | **25.39** |

*Mean values followed by the same letter do not differ statistically at 5% significance according to the Tukey test.
