# Supplementary material for: The times, movements and operational efficiency of mechanized coffee harvesting in sloped areas
Source: PLoS One. 2019 May 28;14(5):e0217286. doi: 10.1371/journal.pone.0217286 (PMC6538159; doi:10.1371/journal.pone.0217286)
Supplement: S4 Table — (DOCX) [file pone.0217286.s008.docx]

**S4 Table. Average of services ha^-1^ of all treatments.**

| **Treatments** | **Services ha^-1^** |
| --- | --- |
| Mechanized (J-FLEX) | 1.51 a |
| Semimechanized (Breaker) | 15.88 b |
| Manual (1 worker) | 35.65 c |
| **Coefficient of Variation (%)** | **5.25** |

* Mean values followed by the same letter do not differ statistically at 5% significance according to the Tukey test.
