# Supplementary material for: The times, movements and operational efficiency of mechanized coffee harvesting in sloped areas
Source: PLoS One. 2019 May 28;14(5):e0217286. doi: 10.1371/journal.pone.0217286 (PMC6538159; doi:10.1371/journal.pone.0217286)
Supplement: S5 Table — (DOCX) [file pone.0217286.s009.docx]

**S5 Table.** **Mean values of the amount of coffee harvested, in L ha^-1^.**

| **Treatments** | **Quantity Harvested (L ha^-1^)** |
| --- | --- |
| Mechanized (J-FLEX) | 11,059.26 a |
| Semimechanized (Breaker) | 14,980.74 b |
| Manual (1 worker) | 15,822.61 b |
| **Coefficient of Variation (%)** | **18.67** |

* Mean values followed by the same letter do not differ statistically at 5% significance according to the Tukey test.
