# Supplementary material for: The times, movements and operational efficiency of mechanized coffee harvesting in sloped areas
Source: PLoS One. 2019 May 28;14(5):e0217286. doi: 10.1371/journal.pone.0217286 (PMC6538159; doi:10.1371/journal.pone.0217286)
Supplement: S6 Table — (DOCX) [file pone.0217286.s010.docx]

**S6 Table. Mean values of the amount of coffee remaining on the plant, in L ha^-1^.**

| **Treatments** | **Coffee remaining** **(L ha^-1^)** |
| --- | --- |
| Mechanized (J-FLEX) | 7760.23 a |
| Semimechanized (Breaker) | 0.00 b |
| Manual (1 worker) | 0.00 b |
| **Coefficient of Variation (%)** | **47.89** |

* Mean values followed by the same letter do not differ statistically at 5% significance according to the Tukey test.
